# The utility of long-term methylphenidate in preserving intellectual development in survivors of childhood brain tumour

**DOI:** 10.1007/s11060-025-05177-9

**Published:** 2025-07-31

**Authors:** Alexander J. Hagan, Rebecca M. Hill, Andrew Kingston, Simon Bailey, Sarah J. Verity

**Affiliations:** 1 School of Medicine, Newcastle University, Framlington Place, Newcastle Upon Tyne, NE2 4HH UK; 2 Department of Paediatric Health Psychology, Paediatric Neuro-Oncology, Great North Children’s Hospital, Royal Victoria Infirmary, Queen Victoria Road, Newcastle Upon Tyne, NE1 4LP UK; 3 Wolfson Childhood Cancer Research Centre, Newcastle University Centre for Cancer, Newcastle Upon Tyne, NE1 7RU UK; 4 Population Health Sciences Institute, Faculty of Medical Sciences, Newcastle University, Campus for Ageing and Vitality, Newcastle Upon Tyne, NE4 5PL UK

**Keywords:** Child, Brain neoplasms, Intelligence, Methylphenidate, Cognitive dysfunction, Neurorehabilitation

## Abstract

**Background:**

Survivors of childhood brain tumour face significant neurocognitive late effects, including impairment in processing speed and attention. Deficits to these core cognitive domains contribute to the plateauing of age-appropriate intellectual development as the child matures. While short-term studies demonstrate the utility of methylphenidate in enhancing processing speed and attention during early recovery, its longer-term role in sustaining intellectual development remains underexplored.

**Methods:**

The current study examined the long-term effects of methylphenidate on intellectual development in 23 survivors of childhood brain tumour, matched on multiple clinical and demographic variables against 23 controls. Intellectual assessments were conducted at baseline and after 12 months of treatment. Statistical analyses evaluated the degree of change in intellectual trajectories between groups over time.

**Results:**

Bayesian hierarchical modelling showed distinct intellectual trajectories between groups, with consistent declines in the control group. Treatment with methylphenidate was associated with the preservation of age-appropriate intellectual development, with the highest posterior probability of treatment-related benefit to Fluid Reasoning (0.97), and strong-to-moderate evidence of benefits to Verbal Comprehension (0.92), Working Memory (0.91), and Processing Speed (0.84). Frequentist analyses supported these findings, demonstrating significant preservation of Fluid Reasoning over time (*t* = 2.14, *p* = 0.04). The use of chemotherapy was associated with a significant decline in Fluid Reasoning across the entire dataset; this effect was only sustained in the control group (*β* = − 18.6, *p* = 0.03).

**Conclusion:**

These findings highlight the long-term rehabilitative potential of methylphenidate and suggest its wider adoption could support care for survivors at risk of intellectual plateau.

**Supplementary Information:**

The online version contains supplementary material available at 10.1007/s11060-025-05177-9.

## Introduction

Brain and spinal tumours are the most common solid malignancies of childhood, accounting for 25% of age-standardised incidence rates in children aged 0–14 years as well as 12% in young adults aged 15–24 years in the United Kingdom [1]. Advancements in molecular classifications, neurosurgical techniques, and targeted adjuvant therapies have led to improvements in survival rates over the last three decades. Survival is however offset by significant iatrogenic morbidity and a diverse spectrum of enduring neurocognitive late effects [2–4].

Current risk-stratified treatment protocols aim to reduce morbidity through the use of novel agents targeting specific oncogenic pathways, alongside the cautious deintensification of conventional therapies [5]. Nevertheless, treatment inevitably exposes the developing brain to varying degrees of neurological insult, hindering age-appropriate cell proliferation, neuronal growth, and myelination [6]. Several demographic, clinical and treatment-related factors have been associated with a greater degree of insult and less favourable neurocognitive outcomes, including younger age at diagnosis [2], the presence of hydrocephalus at diagnosis [7], use of cranial-directed photon radiotherapy [8], and cerebellar mutism syndrome [9]. Advancements in radiotherapeutic techniques, such as proton radiotherapy, offer more precise dose delivery to areas of disease while sparing adjacent healthy tissue [10]. Compared to conventional photon radiotherapy, the use of proton therapy has been associated with more favourable neurocognitive outcomes [10, 11].

### Neurocognitive deficits in childhood brain tumour survivorship

Slowed cognitive processing and attentional difficulties are frequently reported by survivors of childhood brain tumours during the early phases of recovery [12–14]. The reliance of these domains on intricate white matter tracts makes them especially vulnerable to insult, with subsequent impairment having a deleterious effect on higher-order cognitive functioning and knowledge acquisition [14, 15]. Conceptual modelling proposes that deficits in these core cognitive functions (attention, working memory, and processing speed) predict the wider plateauing of age-appropriate intellectual and academic development [14]. The observed decline in age-appropriate intellectual ability often persists for several years after diagnosis resulting in long-term impairment, with the specific rate of decline influenced by various clinical factors such as whole-brain photon radiotherapy, neurotoxic chemotherapy, and hydrocephalus/ventriculoperitoneal shunting [16]. These cognitive deficits in turn contribute to wider systemic difficulties for childhood survivors as they ‘*grow into their difficulties*’, including poorer quality of life [17], social difficulties [18], and poorer mental health [19]. Long-term socioeconomic consequences of brain tumours often present as childhood survivors transition into adulthood, contributing to financial instability, employment struggles, and a decreased likelihood of stable relationships [20].

### The utility of methylphenidate

Psychostimulants have a well-established role in the first-line treatment of childhood Attention Deficit Hyperactivity Disorder (ADHD) [21]. More recently, the use of psychostimulants, most notably methylphenidate, has been extended to attentional deficits secondary to childhood acquired brain injury [22–25]. Despite the relatively small number of published clinical trials, methylphenidate offers promise in enhancing processing speed and attention in survivors of childhood brain tumour during early phases of recovery [22, 26, 27]. Improvements to these core cognitive functions have in turn been associated with improved quality of life and social skills [28, 29].

The proposed mechanism of action involves enhanced dopaminergic and noradrenergic signalling, thereby promoting the functional integrity of large-scale neural networks that underpin attention, working memory and cognitive processing [30]. In survivors of childhood brain tumour, these networks are frequently compromised as a result of both disease- and treatment-related injury to white matter tracts [31]. This provides a plausible neurobiological rationale for methylphenidate treatment, both in enhancing core cognitive functions as well as potentially mitigating downstream effects of disrupted network efficiency.

While short-term studies (2–14 days) demonstrate benefit to core cognitive functions, little is known about the longer-term impact of methylphenidate on broader intellectual outcomes that rely on these foundational domains [22, 26, 27]. Based upon proposed conceptual modelling, we hypothesise that the sustained use of methylphenidate may help preserve age-appropriate intellectual development by enhancing the underpinning functions of attention, working memory, and processing speed [14]. One longitudinal study in childhood ADHD provides partial support for this hypothesis, reporting modest but significant gains in intellectual ability following 12 months of methylphenidate treatment [32].

As such, the current study evaluates the longer-term utility of methylphenidate on intellectual developmental trajectories in survivors of childhood brain tumour over a minimum assessment period of 12 months, compared to a no-treatment control group. While previous research has examined short-term benefits of methylphenidate, and one study has reported non-significant gains to Full-Scale Intelligence Quotient at 12-month follow-up [28], our study is the first to rigorously model longitudinal changes in multiple intellectual domains over sustained treatment, using matched controls and Bayesian analyses to evaluate treatment-related trajectories.

## Methods

### Participants

Eligibility for methylphenidate was determined based upon neurocognitive assessment data gathered as part of routine service provision between April 2017 and April 2023 at the Great North Children’s Hospital, United Kingdom, as previously outlined [25]. In brief, eligible participants for methylphenidate were aged between 5.0–16.5 years at recruitment; were fluent English speakers; had completed treatment for a primary brain tumour with no evidence of recurrent disease; and had a General Ability Index (an estimate of general ability that is not reliant on processing speed or working memory) ≥ 50. Allocation to methylphenidate treatment was typically guided by early post-treatment difficulties in attention and/or processing speed, alongside clinical judgement. Exclusionary criteria included all medical and psychological contraindications to methylphenidate as per NICE Guidelines NG87 [33], and children diagnosed with neurofibromatosis. Forty-two patients met the above eligibility criteria and were prescribed methylphenidate. Of these 42, 23 patients were eligible for the current study, exclusion reasons are detailed in Appendix A.

Eligible patients were matched retrospectively with 23 controls (no methylphenidate) based upon the following demographic and clinical variables; tumour diagnosis, sex, age at diagnosis, presence of hydrocephalus, presence of cerebellar mutism syndrome, and the use of photon or proton radiotherapy. Selected matching variables reflect well-documented clinical risk factors known to contribute to poorer neurocognitive outcomes in childhood brain tumour survivorship [2, 7–11]. While tumour location is recognised to contribute to variability in neurocognitive outcomes, it was not included in the matching process due to the substantial heterogeneity of anatomical sites, the greater clinical relevance of other variables (i.e., tumour diagnosis), and sample size limitations.

### Measures

Tests of intellectual ability were provided using an age-appropriate Wechsler Intelligence Scale; *Wechsler Adult Intelligence Scale—Fourth UK Edition* (WAIS-IV^UK^) [34], *Wechsler Intelligence Scales for Children* (WISC-V^UK^) [35], or *Wechsler Pre-School and Primary Scales of Intelligence* (WPPSI-IV) [36]. Each Wechsler Intelligence Scale consists of 10 core subtests, yielding five primary index scores: Verbal Comprehension, Visual Spatial, Fluid Reasoning, Working Memory, and Processing Speed, except for the WAIS-IV^UK^ which replaces Visual Spatial and Fluid Reasoning with Perceptual Reasoning. Index scores have a mean of 100 and standard deviation of 15. The Wechsler Intelligence Scales demonstrate strong psychometric properties, including high reliability (≥ 0.86) and convergent validity across versions [34–36].

### Procedure

Patients were provided with a baseline intellectual assessment prior to starting methylphenidate. Short acting methylphenidate was provided based on a starting dose of 2.5 mg twice daily for children 15–20 kg; 5 mg twice daily for those 21–30 kg, and 10 mg twice daily for those above 30 kg as per the British National Formulary for Children (BNFC) [25, 37]. Doses were titrated according to clinical response, typically towards 0.3 mg/kg/dose in line with BNFC guidance [37]. Where appropriate, participants were transitioned to an equivalent long-acting formulation. A minority remained on short-acting preparations based on clinical indication. Routine appointments were conducted once every three months to monitor for treatment side effects and complete clinical observations as per NICE Guidelines NG87 [33]. Treatment adherence was reviewed at each routine appointment. All participants included in the final analysis remained on methylphenidate throughout the 12-month follow-up period.

Follow-up intellectual assessments were conducted a minimum of 12 months from baseline assessment. For the control group, intellectual outcome data were retrospectively sourced from case-matched patients, ensuring a minimum of 12 months between assessments.

### Statistical analysis

Index scores^1^ (Verbal Comprehension, Visual Spatial, Fluid Reasoning, Working Memory, and Processing Speed) were collected at baseline and follow-up assessments for both the treatment and control groups. Descriptive analyses were calculated for all intellectual indices. Clinically significant plateauing of intellectual development was defined as a decrease of one standard deviation, or more (i.e., ≥ 15-points), in age-adjusted index scores between baseline and follow-up, based on Wechsler scale norms. A chi-square test of independence was conducted to assess whether the frequency of clinically significant plateauing differed between the treatment and control groups. Two-tailed t-tests were conducted to assess for within-group variation. Of the 460 expected data points across all participants, 58 values were missing, across 49 assessments. Reasons for missingness were predominately due to patient fatigue, limited engagement, or clinician-led decisions to discontinue testing when patients were unable to complete subtests. Data were assumed to be missing at random. This level of missingness was addressed robustly using multiple imputation within each group via the ‘mice’ package, with five imputed datasets generated [38, 39]. Pooled estimates were computed using Rubin’s rules. All statistical analyses were conducted using R version 4.4.2 [40].

Linear Mixed-effects Models (LMMs) with random intercepts were used to assess treatment effects and longitudinal intellectual trajectories using the ‘*lme4*’ package for model specification [41], and the ‘*lmerTest*’ package for hypothesis testing [42]. Model selection was evaluated using Akaike Information Criterion and Bayesian Information Criterion, both indicating that the random intercepts model provided a superior fit compared to alternative models. Assumptions of normality were assessed using the Shapiro–Wilk Test [43]. Additional LMMs were used to examine the influence of clinical and demographic variables on intellectual trajectory. Cohen’s *d* effect sizes were calculated based on model-estimated treatment effects and pooled residual standard deviations.

To complement frequentist analyses, Bayesian linear mixed-effects models were used to assess longitudinal changes in intellectual ability, using ‘*brms*’ package [44]. Models were specified as ‘*Intellectual Score* ~ *Time* + *Gro*up + *Time:Gro*up + *(1 | ID)*’, where *Time* represents assessment point (baseline vs follow-up), and *ID* accounts for random intercepts for each patient. Weakly informative priors followed the ‘*brms*’ package defaults; fixed effects were assigned Normal (0,10) priors, and group-level standard deviations and residual variance were assigned Student_t (3, 0, 10) priors. Models were estimated using Markov Chain Monte Carlo sampling using 4 chains with 4000 iterations each (1000 warm-up), yielding 12,000 total post-warm-up samples. All models showed good convergence (R̂ = 1.00) and adequate effective sample sizes.

Matching was conducted using nearest neighbour propensity scores without replacement via the ‘*MatchIt*’ package, with diagnostics confirming acceptable covariate balance across all matched variables [45].

## Results

Demographic and clinical characteristics of the treatment and control groups are outlined in Table 1. All patients were White British, fluent native English speakers. No significant differences in clinical or demographic variables (i.e., tumour diagnosis) were identified between the treatment and control groups. The mean age at which participants began methylphenidate treatment was 10.8 years (SD = 3.4 years, range = 5–15.7 years). The mean time on methylphenidate at follow-up assessment was 2.1 years (SD = 1.2 years, 1–5.3 years).

**Table 1:** Participant demographic and clinical information, demonstrating no significant between-group differences

	Treatment	Control	P*
	*N*	*N*	
Sex			**1**
Male	19	20	
Female	4	3	
Age at diagnosis (years)			**0.59**
Mean (SD)	5.8 (4.2)	6.4 (4.2)	
Range	0.8–14.6	1.1–15.3	
Age at treatment completion (years)			**0.62**
Mean (SD)	7 (3.8)	7.9 (4.2)	
Range	1.3–14.8	2.1–15.8	
Diagnosis			**0.95**
Medulloblastoma	7	5	
Ependymoma	5	4	
LGG	4	6	
Astrocytoma	3	4	
Germ cell tumour	2	1	
Germinoma	1	1	
Pineoblastoma	1	0	
Choroid plexus papilloma	0	1	
Choroid plexus carcinoma	0	1	
Treatment			**0.90**
Surgery	20	20	
Chemotherapy	15	12	
Photon radiotherapy	13	11	
Proton radiotherapy	5	5	
Comorbidities			**0.36**
Hydrocephalus	16	17	
Ventriculoperitoneal shunt insertion	7	3	
Cerebellar mutism syndrome	5	1	
Age at baseline assessment (years)			**0.61**
Mean (SD)	10.3 (3.3)	11.1 (4.8)	
Range	5–15.6	4–23.2	
Age at follow-up assessment (years)			**0.87**
Mean (SD)	13.1 (3.5)	13.6 (5.9)	
Range	6.3–18	5.1–27.9	

*Mann–Whitney *U* Test or Fisher’s Exact Test

### Clinical and demographic variables

Across baseline and follow-up assessments, younger age at diagnosis was significantly associated with poorer Fluid Reasoning scores (*β* = − 1.40, *p* = 0.04), while older age at treatment was associated with higher Fluid Reasoning scores (*β* = 1.59, *p* = 0.02).

The use of chemotherapy was associated with a significant decrease in Fluid Reasoning scores across the entire dataset (treatment and control groups) at follow-up assessment (*β* = − 15.8, *p* = 0.04). When analysed by group, the negative effect of chemotherapy on Fluid Reasoning scores persisted in the control group (*β* = − 18.6, *p* = 0.03), but was not significant in the treatment group (*β* = 41.8, *p* = 0.18). In the control group, the use of chemotherapy was also associated with significant decrease in Verbal Comprehension scores at follow-up assessment (*β* = − 24.1, *p* = 0.01).

Male sex was associated with significantly poorer Verbal Comprehension scores in the treatment group at follow-up assessment (*β* = − 22.51, *p* < 0.01).

### Intellectual ability

**Fig. 1:**
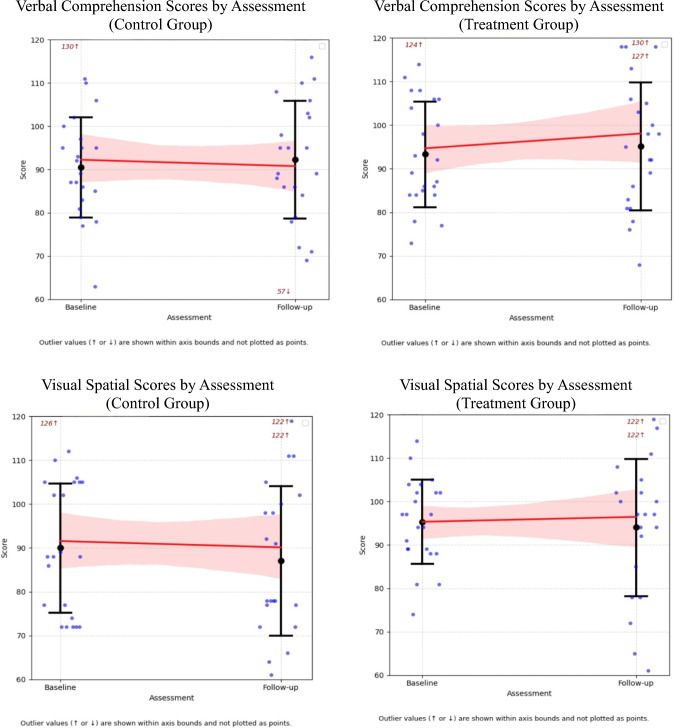

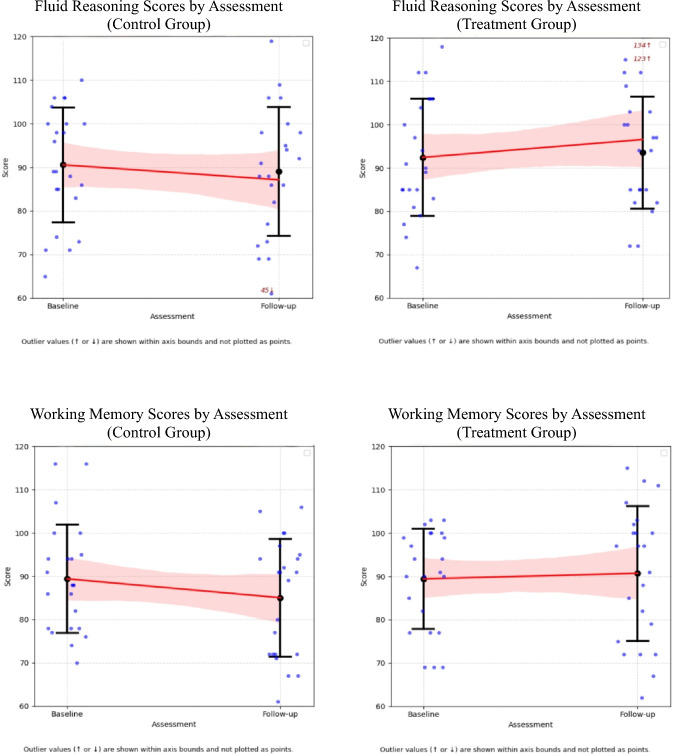

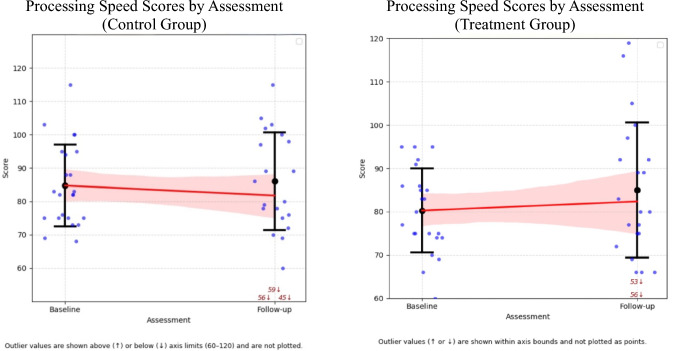
Change in intellectual indices from baseline to follow-up assessments in treatment and control groups

Age-adjusted mean intellectual scores for the treatment and control groups are detailed in Table 2 and illustrated in Fig. 1. Nine index scores (from seven patients) in the treatment group and 16 index scores (from nine patients) in the control group met criteria for clinically significant plateauing, this difference did not reach statistical significance (χ^2^ = 2.20, df = 1 *p* = 0.14). Descriptively, these individuals did not differ meaningfully in age, sex, tumour type, or treatment exposure. No significant within-group differences in intellectual performance were reported between baseline and follow-up for either the treatment or control groups based on paired t-tests.

**Table 2:** Differences between baseline and follow-up and mean change values

	Treatment group					Control group						Mean change^b^			
	Baseline	SE	Follow-up	SE	*p*^*a*^	Baseline	SE	Follow-up	SE	*p*^*a*^	Difference	SE	95% CIs^c^	*d*^*d*^	*p*
Verbal comprehension	94.1	2.9	98.0	3.5	0.22	92.2	2.9	90.7	3.2	0.59	4.77	3.6	[− 2.27, 11.81]	0.56	**0.19**
Visual spatial	96.3	2.1	94.1	3.5	0.55	92.9	5.7	84.8	4.9	0.10	6.46	4.6	[− 2.54, 15.46]	0.77	**0.17**
Fluid reasoning	90.8	3.7	96.6	3.3	0.22	90.8	2.9	87.1	3.7	0.13	8.53	4.0	[0.73, 16.33]	0.98	**0.04**
Working memory	91.6	2.5	91.3	3.4	0.47	89.6	3.0	85.1	3.2	0.07	5.58	3.7	[− 1.70, 12.87]	0.74	**0.14**
Processing speed	80.3	2.0	82.4	3.6	0.49	85.3	2.8	81.1	3.8	0.74	4.40	4.1	[− 3.67, 12.48]	0.46	**0.29**

^a^*P* value from paired sample t-tests examining within-group differences between baseline and follow-up

^b^Estimated mean change difference between the treatment and control groups from baseline to follow-up, derived from linear mixed modelling

^c^95% confidence intervals were calculated using normal approximation (linear mixed model estimate ± 1.96 × standard error)

^d^Cohen’s* d* effect sizes were calculated based on model-estimated treatment effects and pooled residual standard deviations

Linear Mixed Models (LMM) assessing group differences over time reported a significant improvement in Fluid Reasoning for the treatment group compared to the control group (*t* = 2.14, *p* = 0.04, Table 2). A second supplementary LMM, which assessed whether the rate of change in intellectual scores over time differed between groups, reported a similar significant treatment effect on Fluid Reasoning (*p* = 0.03, Appendix B).

Bayesian hierarchical modelling revealed distinct intellectual trajectories between treatment and control groups (Table 3). While the control group demonstrated consistent declines across all intellectual domains, the treatment group exhibited preservation of age-appropriate intellectual development over time. The probability of treatment-related benefit was highest in Fluid Reasoning (0.97), with strong-to-moderate evidence of benefits to Verbal Comprehension (0.92), Working Memory (0.91), and Processing Speed (0.84), while Visual Spatial demonstrated the weakest treatment response (0.76).

**Table 3:** Bayesian model findings for intellectual performance trajectories

	Estimated baseline score (control)	Change over time (control)	Change over time (treatment)	Difference in change (treatment vs control)	Posterior probability (Effect > 0)
Verbal comprehension	92.1 [86.3, 97.9]	− 1.2 [− 5.9, 3.6]	+ 3.2 [− 2.1, 8.9]	4.4 [− 2.3, 10.8]	**0.92**
Visual spatial	91.5 [85.3, 97.7]	− 1.3 [− 6.1, 3.6]	+ 1.1 [− 5.1, 7.5]	2.3 [− 4.2, 8.9]	**0.76**
Fluid reasoning	90.3 [84.3, 96.2]	− 2.9 [− 7.5, 1.8]	+ 3.8 [0.3, 9.0]	6.6 [0.3, 12.9]	**0.97**
Working memory	89.1 [83.8, 94.4]	− 3.9 [− 8.4, 0.5]	+ 1.0 [− 4.9, 7.4]	4.9 [− 1.3, 11.0]	**0.91**
Processing speed	84.0 [78.1, 89.9]	− 2.3 [− 7.7, 3.2]	+ 1.6 [− 4.1, 8.0]	3.9 [− 3.6, 11.4]	**0.84**

Posterior means are shown with 95% credible intervals in square brackets. Posterior probabilities reflect the probability that the treatment effect is greater than zero

## Discussion

The long-term neurocognitive late effects of childhood brain tumour remain a significant clinical concern, with early deficits in processing speed and attention contributing to the plateauing of intellectual development over time. While previous research has demonstrated short-term benefits of methylphenidate following 2–14 days of treatment [22, 26, 27], its extended utility in preserving intellectual development remains largely overlooked. The current study provides novel evidence of the rehabilitative potential of methylphenidate in stabilising intellectual development over a minimum treatment period of 12 months. These findings support the integration of methylphenidate into standard paediatric neuro-oncology care pathways to ensure equitable access to neurorehabilitation for those at high risk of long-term neurocognitive impairment.

### Intellectual Development

Bayesian analysis indicate a high likelihood that methylphenidate contributes to the preservation of age-appropriate intellectual development, offsetting the progressive decline observed in controls and leading to fewer childhood survivors meeting criteria for clinically significant plateauing of intelligence. The sustained benefit across intellectual domains aligns with the proposed neurobiological mechanism of methylphenidate, which enhances dopaminergic and noradrenergic signalling across multiple brain networks involved in core cognitive functions [30]. This broad enhancement may buffer declines in domains reliant on neural efficiency (e.g., processing speed) and prevent the plateau of broader cognitive functions, thereby preserving age-adjusted intellectual development [2, 4, 31].

As a substantial proportion of patients likely had posterior fossa tumours (i.e., medulloblastoma and ependymoma), it is plausible that these patients also experienced some degree of cerebellar dysfunction, which is associated with a spectrum of higher-order cognitive deficits [46]. Despite these diffuse cognitive vulnerabilities, the observed benefits suggest that methylphenidate may exert broad modulatory effects that also support functional recovery across distributed cerebro-cerebellar circuits, contributing to the preservation of intellectual development.

Given the extensive cognitive benefits of methylphenidate, its extended use may therefore promote systemic functional benefits as childhood survivors reintegrate into cognitively and socially demanding environments, such as supporting improved academic attainment, enhancing peer relationships, and promoting quality of life [8, 17, 18].

### Fluid Reasoning

Fluid reasoning is a multifaceted intellectual domain encompassing abstract reasoning and novel problem solving, playing a central role in adaptive learning during childhood [35]. As a relatively recent addition to the Wechsler scales, fluid reasoning remains understudied in survivors of childhood brain tumour, with earlier research primarily focused on working memory and processing speed due to scale limitations and their known clinical relevance [2, 12, 22]. Emerging neuroimaging research in children born extremely preterm links fluid reasoning to frontoparietal white matter integrity [47]. These findings align with broader neurodevelopmental models positing that intact frontoparietal white matter connectivity (particularly involving the superior longitudinal fasciculus and frontoparietal projections) are critical for the development of robust abstract reasoning, cognitive flexibility and fluid reasoning [48]. Neuro-oncological treatments, including chemotherapy and radiotherapy, disrupt white matter development by damaging oligodendrocyte precursor cells essential for myelination and network maturation, which likely impacts fluid reasoning development in survivors [49].

Fluid reasoning demonstrated the greatest degree of preservation with methylphenidate, supported by both Bayesian and frequentist analyses. Increased dopamine and noradrenaline in prefrontal and striatal regions likely act to enhance executive functions underpinning fluid reasoning [50]. Methylphenidate has also been associated with increased white matter fractional anisotropy in several association tracts of the left hemisphere and the lateral aspect of the corpus callosum with longer-term use (16 weeks) [51]. This may facilitate more efficient neural communication across brain regions and help prevent the plateauing of intellectual development.

### Clinical variables

Chemotherapy was the strongest predictor of intellectual impairment, associated with significant decreases in fluid reasoning across the entire dataset. When examined by group, the detrimental effect of chemotherapy on fluid reasoning persisted in the control group, but was attenuated to a non-significant level in the treatment group. Additionally, chemotherapy was associated with a significant decrease in verbal comprehension scores in the control group at follow-up assessment. These findings suggest that methylphenidate may help buffer or reduce the neurotoxic effect of chemotherapy on vulnerable cognitive domains, such as fluid reasoning [2]. While the current study focused on survivors of childhood brain tumour, these findings may extend to other paediatric groups with similar neurocognitive difficulties, most notably traumatic brain injury, highlighting the broader rehabilitative potential of methylphenidate.

Male sex was associated with significantly poorer verbal comprehension ability at follow-up assessment in the treatment group. While this finding should be interpreted with caution due to the modest sample size, it aligns with a similar sex-based difference in verbal comprehension reported in a Dutch cohort [52]. Authors from this previous study attributed the sex difference to larger tumour size at diagnosis in males, particularly among those presenting with raised intracranial pressure [52]. As tumour size was not controlled for in our analysis, this may partially account for the observed effect. Nevertheless, this finding should be considered exploratory, and future research with larger cohorts is warranted to clarify whether sex moderates verbal comprehension recovery following treatment.

Of note, five patients within the treatment group were diagnosed with cerebellar mutism syndrome (CMS), a postoperative neurological condition characterised by long-term deficits in language-related domains, including impairments in verbal fluency and expressive syntax [9, 46]. In contrast, only one patient in the control group was diagnosed with CMS. As verbal comprehension subtests are particularly sensitive to such deficits, the relatively large CMS subgroup, of whom four were male, may have contributed to lower scores in this domain at follow-up. Although male sex was statistically associated with poorer verbal comprehension scores, the presence of CMS within this subgroup may represent a more proximate explanatory factor. While our matching strategy accounted for several clinical and demographic variables, survivors of childhood brain tumour are an inherently heterogeneous clinical group, limiting the feasibility of complete adjustment for all potential confounds.

### Clinical implications

By preserving age-appropriate intellectual trajectories, methylphenidate may help mitigate the long-term impact of disease- and treatment-related neurocognitive deficits, which predispose survivors to academic underperformance, social isolation, and reduced quality of life. Integrating methylphenidate into neurorehabilitation pathways, alongside proactively identifying patients at risk of intellectual decline, can support earlier clinical decision-making and targeted support for childhood survivors at the greatest risk. This proactive approach aligns with a broader shift toward survivorship care models that prioritise quality of life alongside survival. To maximise clinical impact and promote ethical implementation, future integration of methylphenidate into neurorehabilitation pathways should ensure equitable access and incorporate the lived experiences of childhood survivors and their families.

### Future directions

Future research should replicate these findings in larger, diverse cohorts and evaluate long-term outcomes, including academic achievement and mental health. Addressing variability in access to methylphenidate and neurocognitive rehabilitation services is critical to promote equity in survivorship care. A coordinated multi-centre approach is needed to assess generalisability and feasibility across clinical contexts.

We acknowledge that maturational stage may moderate neurocognitive recovery and treatment effects beyond chronological age alone. While the current sample was underpowered to investigate developmental timing directly, one of the authors (SJV) is a collaborator on a preclinical mouse modelling study examining precisely this. There are also plans to explore this further with a wider human cohort, comparing the impact of methylphenidate immediately post-treatment versus at 2 years post-treatment.

Additionally, future studies should incorporate patient and parent perspectives to ensure survivorship interventions align with the lived experiences and priorities of families. The integration of pharmacological rehabilitation into standard of care has the potential not only to improve individual outcomes but also to yield broader societal benefits. By preserving cognitive function, such interventions are likely to support more successful reintegration into education and future employment, particularly for those survivors at risk of long-term functional disadvantage. Combining pharmacological interventions, such as methylphenidate, with structured cognitive training may offer additive benefits for survivors, and represents a promising future direction for enhancing long-term neurocognitive outcomes in childhood brain tumour survivorship.

### Limitations

Strengths of this study include robust statistical methods, broad eligibility criteria, and comprehensive participant matching using nearest neighbour propensity score strategies, shown in simulations to minimise bias and maximise precision. However, limitations include the lack of data on socioeconomic status and limited ethnic diversity, which may affect the generalisability of these findings to more ethnically and geographically diverse populations. The use of retrospective controls, non-randomised group allocation, and absence of blinding introduce the risk of selection and performance biases. Despite rigorous matching, residual confounds may persist due to tumour heterogeneity, including unaccounted variability in tumour anatomical location which may influence neurocognitive outcomes. The study may also be underpowered to detect subtle group differences or specific interaction effects. Random slopes were excluded due to convergence and model complexity concerns. Objective measures of adherence were not available, and while occasional nonadherence cannot be ruled out, it is unlikely to have meaningfully affected the validity of our findings. Additionally, different versions of the Wechsler Intelligence Scales were used based on participant age without cross-scale norm equivalence procedures. While minor structural differences between scales may have introduced some measurement variability, the high reliability and convergent validity across different scales support the comparability of index scores and is unlikely to have meaningfully affected our overall findings.

## Conclusion

This study highlights the long-term benefits of methylphenidate in stabilising intellectual development among survivors of childhood brain tumour. These findings strongly support its integration into standard care pathways to mitigate cognitive impairments and enhance quality of life. Guided by patient and parent input and evaluated through multicentre collaboration, this approach has the potential to preserve neurocognitive function and improve long-term educational, occupational, and societal outcomes for survivors of childhood brain tumour.

## Supplementary Information

Below is the link to the electronic supplementary material.

Supplementary file1 (DOCX 19 KB)

## Data Availability

The anonymised data that support the findings of this study are available from the corresponding author upon reasonable request.

## Footnotes

1 For the purpose of this study and due to the modest sample size, Perceptual Reasoning calculated by the WAIS-IV^UK^ was pooled with Fluid Reasoning calculated by the WISC-V^UK^ and WPPSI-IV. Correlational analyses evaluating the Standard Difference of the two test means (Fluid Reasoning vs Perceptual Reasoning) is reported as < 0.00 [35].
