# Supplementary material for: The utility of long-term methylphenidate in preserving intellectual development in survivors of childhood brain tumour
Source: J Neurooncol. 2025 Jul 31;175(2):801–12. doi: 10.1007/s11060-025-05177-9 (PMC12420733; doi:10.1007/s11060-025-05177-9)
Supplement: Supplementary file 1 — Supplementary file1 (DOCX 19 KB) [file 11060_2025_5177_MOESM1_ESM.docx]

**Appendices**

**Appendix A**

Table S1. Reasons for Participant Exclusion

| **Reason for Exclusion** | **Number of Participants** | **Details** |
| --- | --- | --- |
| No follow-up intellectual assessment data available | 8 |  |
| Methylphenidate treatment duration less than 12 months at time of writing | 8 |  |
| Discontinuation of methylphenidate within the first 12 months | 3 | Potential development of seizures (n = 1) |
|  |  | Significant negative impact on mood (n = 1) |
|  |  | No perceived benefit as reported by parents (n = 1) |

**Appendix B**

Table S2. Linear Mixed Model – Fixed Effects

|  | **Fixed Effect** | **Estimate (β)** | **Std. Error** | **t-value** | ***p* value** |
| --- | --- | --- | --- | --- | --- |
| **Verbal Comprehension** | Time | -1.48 | 2.50 | -0.59 | **.56** |
|  | Time x Group | 4.87 | 3.53 | 1.38 | **.18** |
|  | Group Difference | 2.44 | 4.41 | 0.55 | **.58** |
| **Visual Spatial** | Time | -1.44 | 2.53 | -0.57 | **.57** |
|  | Time x Group | 2.57 | 3.58 | 0.72 | **.48** |
|  | Group Difference | 3.78 | 4.70 | 0.81 | **.42** |
| **Fluid Reasoning** | Time | -3.44 | 2.42 | -1.42 | **.16** |
|  | Time x Group | 7.57 | 3.43 | 2.21 | **.03** |
|  | Group Difference | 1.87 | 4.44 | 0.42 | **.68** |
| **Working Memory** | Time | -4.35 | 2.30 | -1.89 | **.07** |
|  | Time x Group | 5.61 | 3.26 | 1.72 | **.09** |
|  | Group Difference | 0.04 | 3.95 | 0.01 | **.99** |
| **Processing Speed** | Time | -2.96 | 2.86 | -1.03 | **.31** |
|  | Time x Group | 5.04 | 4.05 | 1.25 | **.22** |
|  | Group Difference | -4.48 | 4.33 | -1.03 | **.31** |
